# Thyroid Cancer: Molecular Aspects and New Therapeutic Strategies

**DOI:** 10.1155/2012/847108

**Published:** 2012-07-12

**Authors:** Enrique Grande, Juan José Díez, Carles Zafon, Jaume Capdevila

**Affiliations:** ^1^Department of Medical Oncology, Ramón y Cajal University Hospital, 28034 Madrid, Spain; ^2^Department of Endocrinology, Ramón y Cajal University Hospital, 28034 Madrid, Spain; ^3^Department of Endocrinology, Vall d'Hebron University Hospital, 08035 Barcelona, Spain; ^4^Department of Medical Oncology, Vall d'Hebron University Hospital, 08035 Barcelona, Spain

## Abstract

Despite that thyroid cancer accounts for over 90% of tumors that arise from the endocrine system, these tumors barely represent 2% of solid tumors in adults. Many entities are grouped under the general term of thyroid cancer, and they differ in histological features as well as molecular and clinical behavior. Thus, the prognosis for patients with thyroid cancer ranges from a survival rate of >97% at 5 years, in the case of differentiated thyroid tumors sensitive to radioactive iodine, to a 4-month median survival for anaplastic tumors. The high vascularity in these tumors and the important role that oncogenic mutations may have in the RAS/RAF/MEK pathway and oncogenicity (as suggested by activating mutations and rearrangements of the *RET* gene) have led to the development of multitarget inhibitors in different histological subgroups of patients. The correct molecular characterization of patients with thyroid cancer is thought to be a key aspect for the future clinical management of these patients.

## 1. Introduction

Three different types of thyroid cancer have been defined according to their histological features: differentiated thyroid cancer (DTC) deriving from epithelial cells from the thyroid follicles; medullary thyroid cancer (MTC); anaplastic thyroid cancer (ATC). Approximately 90% of diagnosed thyroid cancers correspond to DTC, with papillary (PTC) histology being the most frequent (75%), followed by follicular (FTC) (10%), Hürthle cells (5%), and poorly differentiated carcinomas (1–6%) [1, 2]. Overall, MTC accounts for approximately 10% of all thyroid tumors and ATC barely 1% [2].

Thyroid cancer is considered a nonfrequent entity because it represents only 1% of all solid tumors in adults. Women are three-times more likely to suffer this disease, and the incidence of DTC has increased 2.4-fold over the past 30 years [3]. Most DTC patients have a very good prognosis if diagnosed at early stages, and 91% of patients are alive at 20 years when the classical treatment with surgery followed by radioiodine ablation and suppression of thyroid stimulating hormone (TSH) is employed. However, patients with DTC resistant to radioactive iodine, or those with MTC or ATC, have limited options for treatment. Up to now, the chemotherapy drugs most often used worldwide to treat thyroid cancer are doxorubicin and cisplatin. However, the most referenced guidelines in the field do not always recommend the routine use of these agents in the clinic. Doxorubicin and cisplatin showed some activity in a study conducted in the 1970s in a total of 92 patients with all types of thyroid cancer; a progression-free survival of 3 months; an overall survival of 7 months being achieved following treatment [4]. Currently, the use of chemotherapy in patients with thyroid cancer is limited to patients with ATC, those with poorly differentiated histology with a high rate of proliferation, or in highly symptomatic patients who are not candidates for other local or systemic therapies.

The last decade has seen advances in the molecular biology that may underlie the development and progression of these tumors. For example, DTC is mainly associated with mutations in the RAS/RAF/MAPK intracellular signaling pathway or with gene rearrangements such as *RET/PTC*. Virtually all MTC tumors associated with hereditary syndromes and about 45% of sporadic MTC are associated with mutations activating the *RET* gene [5].

Classically, thyroid tumors are associated with high vascularization and high levels of vascular endothelial growth factor, fibroblast growth factor, and platelet-derived growth factor (VEGF, FGF, and PDGF, resp.) [6]. The importance of angiogenesis in these neoplasms became clear when several studies showed that tumors with high levels of VEGF in the stroma correlated with a greater propensity towards metastases at distance, as well as with other markers of tumor aggressiveness. Of considerable note is that thyroid cell cultures have a reduced proliferation when the VEGF pathway is blocked, thus demonstrating a direct antitumor activity of antiangiogenic drugs on thyroid tumor cells [7]. Additionally, VEGFR3 is involved in the lymphangiogenesis process, that is, of special interest in papillary thyroid carcinomas that commonly metastasizes in regional lymph nodes [8].

Tyrosine kinases are enzymes that transfer phosphate groups from adenosine triphosphate (ATP) to tyrosine residues of another protein. Many tyrosine kinases are documented in humans as being involved in key processes of cellular control such as survival, proliferation, differentiation, function, and cell motility. In recent years, a large group of low molecular weight agents capable of inhibiting the function of tyrosine kinases have changed the natural history and management of various solid tumors such as kidney cancer, liver cancer, gastrointestinal stromal tumor (GIST), and, more recently, pancreatic neuroendocrine tumor. These drugs are called multitarget tyrosine kinase inhibitors and, due to their biochemical structure being similar to that of ATP, they are able to block the intracellular activation of several membrane receptors or proteins in the intracellular signaling cascade with tyrosine kinase activity. The degree of affinity and selectivity of this inhibitory activity is very variable (Table 1) [6].

Advances in understanding the molecular pathogenesis of different subtypes of thyroid cancer have, currently, made this field of research one of the busiest in the world of endocrine oncology. The application of discoveries in the laboratory to the patient (the “bench-to-bed” transfer) is one of the biggest challenges in translational research area, since these tumors can become a paradigm of therapy based on molecular features that govern the development and growth of tumors in each individual patient. This paper aims to summarize the key molecular determinants of each histological subtype of thyroid cancer and the data derived in recent years from clinical studies conducted with multitarget agents.

## 2. Differentiated Thyroid Cancer (DTC)

### 2.1. Clinical Management of DTC Patients Sensitive to Radioactive Iodine

DTC represents the vast majority of thyroid tumors. Treatment is based on thyroidectomy with or without lymphadenectomy regarding papillary or follicular histology, followed by radioiodine ablation in those patients at high risk of lymph node metastases and levothyroxine in doses that suppress TSH levels. The semiannual or annual monitoring using cervical ultrasound, measurement of thyroglobulin levels as a tumor marker, and whole-body iodine scan is appropriate for these patients.

It is estimated that between 10 and 15% of patients undergoing DTC will have disease relapse. In 3 out of 4 cases, recurrence occurs at the cervical level in the thyroid bed or lymph node level. In these patients salvage surgery, radioiodine therapy and external radiotherapy is recommended in highly selected patients.

Distant metastases occur in <10% of DTC patients, and only half of them are apparent at the time of diagnosis. The lungs are the main receptor organs of these tumor metastases (50%), followed by bone (25%). Initial treatment of patients with disseminated disease includes TSH-suppressive doses of levothyroxine plus radioiodine administered in conditions of high serum TSH levels in those patients with positive total body scan uptake, albeit complete remission is achieved in only 30% of subjects [9].

### 2.2. Clinical Management of Patients with Radioactive Iodine-Refractory DTC

A patient is considered resistant to the use of radioactive iodine when at least one tumor lesion is observed that does not show uptake of radioiodine, or when the lesion radiologically progresses in the first 12 months post-radioiodine administration, or when the patient has persistent disease following the administration of an accumulated dose of radioactive iodine of >600 mCi [10]. The median survival of patients with radioiodine-resistant DTC and distant metastases ranges between 3 and 6 years. It is in these patients that the application of new multitarget inhibitors has its main testing ground [8]. The use of classical chemotherapy, both as monotherapy and in combination with other treatments, in patients with radioiodine-refractory DTC has not shown any significant benefit in these patients and, conversely, has shown high toxicity. The most recent study conducted with doxorubicin was published in 2008 by Matuszczyk et al. [11]. Among the 22 recruited patients with radioiodine-refractory DTC, only one (5%) achieved a partial positive response that lasted for 6 months. Adverse effects included alopecia (42%), nausea (23%), pneumonia (20%), and neutropenia (10%). Of the 22 patients, 12 showed disease progression during the first measurement of treatment response, and only 42% of the patients recruited for the trial had stable disease for a median of 7 months. Other authors have also tested several combination regimes with specific chemotherapy agents such as taxanes, platinum, or gemcitabine, with little added clinical benefit; response rates ranging between 0 and 22% and without an amelioration of progression-free survival (PFS) of the disease [12].

### 2.3. Molecular Biology of DTC (Figure 1)

In recent years, several different genetic events have been identified as being related to the genesis of the DTC. Further, a correlation has been reported between the manifestation of histological alterations of the disease and the presence of changes in the regulation of intracellular molecular pathways that confer a distinct clinical behavior. Two major molecular determinants that involve the same molecular pathway are responsible for the appearance of a PTC: the alteration of gene regulation of the *RET* gene expression and the abnormal activation of signaling pathways, RAS/RAF/MAPK. There is an increasing body of evidence showing that the role of PI3K/Akt/mTOR pathway may be crucial for tumor development and may become an attractive target in the future [13].

The abnormal chromosome rearrangement of proto-oncogene *RET* (rearranged during transfection) with the tyrosine kinase domain of other genes located in the same or other chromosomes results in the fusion oncogene *RET/PTC* that plays a key role in the pathogenesis of up to 30% of patients with PTC [14]. Their involvement is even greater in childhood PTC and in patients whose tumor results from exposure to ionizing radiation (approximately 50–80% of cases). The *RET* proto-oncogene is activated by fusion of the RET TK domain with the 5′-terminal sequence of one of different heterologous genes via rearrangements that generate a series of chimeric-transforming oncogenes collectively described as *RET/PTCs*. The results of *RET/PTC* reorganization are abnormal proteins constitutively activated from RET kinase, independently of the binding to extra-cellular ligands, that activate multiple intracellular signaling pathways leading to clonal expansion and neoplastic transformation of the thyroid follicle cells. It is widely known that *RET* oncogene rearrangements plays a crucial role in radiation-associated papillary thyroid carcinogenesis. Other proto-oncogene rearrangements have been identified affecting to the *NTRK1* gene in PTCs. The frequency of these rearrangements could be seen in up to 25% of patients with PTC. *NTRK1* proto-oncogene rearrangements result in the formation of chimeric genes composed of the tyrosine kinase domain of NTRK1 fused to 5′ sequences of different genes (*TPM3, MET*, and *TRK-T2*). It is estimated that *NTRK1* proto-oncogene is activated by rearrangement with a similar frequency in “spontaneous” and radiation-associated thyroid tumours. Moreover, *NTRK1* proto-oncogene activating rearrangements play a role in the development of a minority of radiation-associated PTC but not in adenomas [15].

Molecular aberrations observed in FTC are significantly different from those described in PTC, including RAS mutations in up to 45% of patients and rearrangements involving the transcription factor gene of the paired-box 8 *(PAX8)* and peroxisome proliferator-activated receptor-*γ* *(PPAR*γ*)* resulting in a *PAX8/PPAR*γ** rearrangement observed in up to 35% of FTC [12].

Genetic alterations in the signaling pathway of RAS/RAF/MAPK constitute the most frequent genetic/molecular anomaly in patients with DTC. BRAF is a serine-threonine kinase that is key in the intracellular regulation of this cellular pathway. Activating mutations have been observed in the V600E codon of the *BRAF* gene in up to 70% of patients with PTC. Similarly, patients with mutations in *BRAF* have more chances of developing resistance to radioactive iodine resulting from alterations in the normal patterns of mobilization and metabolism of iodine in the thyroid follicle cell. These features confer poorer clinical prognosis to patients carrying the mutation. The *V600E BRAF* activating mutation is associated with a loss of expression of the unidirectional cotransporter (symport) sodium iodine and, consequently, a lower uptake of radioiodine by the metastatic lesions of tumors that harbor the mutation. This decreased expression of symport is mediated via the TGF*β* autocrine pathway.

Point mutations in the gene encoding the *RAS* oncoprotein occur to a lesser extent (15%) in PTC, but their frequencies are increased in patients with FTC and less differentiated histologies. Mutations in any gene encoding for *RAS* (*HRAS*, *KRAS* and *NRAS*) involve abnormal activation of mitogen-activated protein kinase (MAPK) and the PI3K/Akt/mTOR alternative parallel route [12, 33].

Mutations in *BRAF*, *RAS* and *RET* rearrangements are involved in >70% of PTC. However, these molecular alterations are mutually exclusive in most cases and are rarely expressed simultaneously within the same tumor. As such, they are independently able to promote PTC.

**Figure 2:**
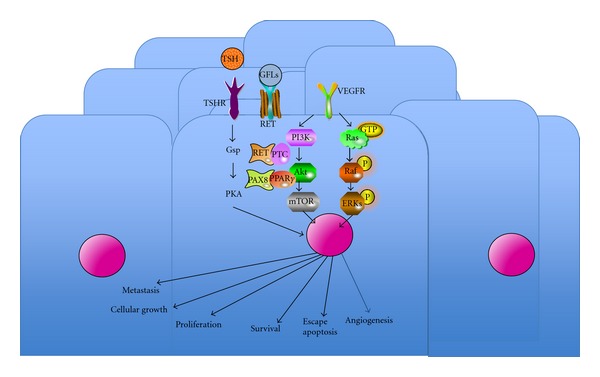
Schematic representation of the follicular tumor cell.

Activation and dysregulation of the intracellular signaling cascade PI3K/Akt/mTOR through loss of expression of tumor suppressor phosphatase PTEN has been implicated in the development of DTC and undifferentiated tumors of the thyroid. Likewise, mutations in the gene *PI3CA*, encoding for the phosphatidyl-inositol-tri-phosphate kinase (PI3K), play a role as initial triggers that activate the intracellular signaling pathway (Figure 2).

Another molecular alteration frequently found in DTC is amplification of the gene encoding for the hepatocyte growth factor (*c*-MET) receptor. The MET membrane receptor cooperates with VEGF receptor to promote angiogenesis, cell mobility, and survival of thyroid cells. Amplification occurs in 80% of papillary thyroid tumors, while its silencing in animal models induces tumor regression, reduction in tumor size, and the appearance of new distant metastases [12].

Angiogenesis is another potential area for the design of new molecular targets. Known receptors for VEGF (VEGFR-1, -2, and -3) and the FGF (FGFR) and PDGF (PDGFR-*α* and -*β*) have been shown to be associated with an increased risk of distant metastases, an increased risk of disease recurrence, less protracted PFS, and increased presence of mutations in BRAF. In *in vitro* experimental models, blockade of the VEGF pathway has succeeded in delaying DTC growth [10].

### 2.4. Multitarget Agents in Current Clinical Development for the Treatment of DTC (Table 2)

#### 2.4.1. Sorafenib

Sorafenib is a multitarget agent for several major molecular pathways involved in the development and progression of PTC, including the serine-threonine kinase BRAF and the tyrosine kinases from the membrane receptors VEGFR-2 and PDGFR-*β* (Table 1). From a biological standpoint, the action profile of sorafenib is the most complete in terms of activity on key molecular targets involved in PTC. Data are available from at least 4 nonrandomized phase II studies which used a dose of sorafenib 800 mg/day as a single agent in patients with DTC refractory to radioactive iodine. In the 30 patients treated by the group of Gupta-Abramson et al. in Philadelphia, a median PFS of 18.4 months was achieved; 7 (23%) patients achieving an objective radiological partial response and 16 patients (53%) achieving disease stabilization of >6 months. Those patients with BRAF mutation (*n* = 16) achieved a median PFS of 84 weeks versus 54 weeks for those who did not have this mutation [16]. In a more recent study, also conducted in the USA, similar results were observed in 41 patients with PTC. In these patients, the objective radiological response rate was 15%, and disease stabilization was observed in 56% of patients. The median PFS was 15 months; the presence of BRAF mutation was observed in 17 (77%) of the 22 patients analyzed [17]. In the UK Matisse study (Safety and Efficacy of Sorafenib in Advanced Metastatic Thyroid Cancer), a total of 30 patients with thyroid cancer were treated, up to 14 with DTC. The objective response rate was 18% [18]. In a more recent study conducted in 31 patients, the response rate achieved was 24%, with a median PFS of 14 months [19]. Most adverse effects occurring in these 4 studies were consistent with the already-known safety profile of the drug; the majority of toxicities found were grade I and II and easily manageable with a delay or dose reduction of sorafenib administration. The data from a retrospective analysis of 34 patients with refractory thyroid cancer treated with sorafenib in Spain have recently been published. Clinical benefit of sorafenib was 73% with a response rate in DTC of 19% and 47% in MTC [20]. Taken together, these results formed the scientific basis for the launch of a phase III registration. Termed DECISION (Study of Sorafenib in Metastatic or Locally Advanced, Refractory Patients with Thyroid Cancer RAI), the study compared the administration of sorafenib versus placebo in 380 patients with radioiodine-refractory DTC with PFS as the primary endpoint (NCT00984282). This study has just completed recruitment, and results are awaited with interest.

#### 2.4.2. Vandetanib

Vandetanib (ZD6474) is a low molecular weight inhibitor of tyrosine kinases, which acts mainly against VEGFR-2, VEGFR-3, and RET and, in higher concentrations, on the epidermal growth factor receptor (EGFR) (Table 1). Vandetanib was one of the first drugs to demonstrate activity in thyroid cancer cell lines, mainly through its action on the rearrangements *RET/PTC* and on RET mutations. Vandetanib has been the subject of report of the largest randomized clinical trial conducted to date in patients with DTC refractory to radioiodine. A total of 145 patients received vandetanib at doses of 300 mg/day or placebo. PFS, which was the primary endpoint, was significantly higher from the clinical as well as statistical point of view, in patients receiving the study drug (11 months) as compared to those receiving placebo (5.8 months) with a relative risk (hazard ratio) of 0.63 (95% CI: 0.43–0.92). However, the objective response rate was less than 5% in the vandetanib arm, and is difficult to explain. The most frequent adverse events found in the vandetanib arm were diarrhea (74%), asthenia (26%), fatigue (23%), nausea (25%), hypertension (34%), hyporexia (26%), and skin rash (25%). Updates from this study are expected in the near future [21].

#### 2.4.3. Axitinib

Axitinib is one of the most selective and potent tyrosine kinase inhibitors currently under clinical development. The main targets blocked by axitinib are VEGFR-2, PDGFR-*β*, and c-KIT, in the subnanomolar range (Table 1). In a prospective noncontrolled phase II trial, axitinib was the first drug that demonstrated clinical activity by inducing objective radiological response in all histological subtypes of thyroid cancer (DTC, MTC, and ATC). Of a total of 60 patients who entered the study, 37 patients had DTC refractory to radioactive iodine; 15 of 47 (31%) patients achieved an objective response rate (by RECIST criteria); 20 (42%) had disease stabilization beyond 6 months when treated with 5 mg of axitinib every 12 hours as a single agent. The median PFS was 18 months, if all histologies were included. Of note is the finding that the median time on treatment with the drug was only 4.8 months, especially considering the high median PFS achieved. The most common adverse events found were asthenia, diarrhea, nausea, anorexia, hypertension, and mucositis [22].

#### 2.4.4. Sunitinib

Sunitinib is a multitarget inhibitor of 3 known receptors of VEGF, as well as of RET and PDGFR-*α* and -*β*, and of the protein derived from the *RET/PTC* realignment (Table 1). In a phase II study [23], sunitinib, administered at a dose of 37.5 mg/day in continuous schedule, induced metabolic complete response in 28 DTC patients, with uptake of distant disease on PET/CT scan a partial response in 7 cases (25%), and disease stabilization in 14 (50%). The median time to progression (TTP) was 12.8 months, and the decline in the uptake of fluorodeoxyglucose (FDG) at 7 days of treatment with sunitinib was superior in those patients who subsequently achieved positive radiological response (by RECIST criteria). In another phase II study conducted in 31 evaluable patients with DTC with metastatic disease progression, sunitinib administered at a dose of 50 mg/day for 4 weeks followed by 2 weeks off treatment, achieved a partial response rate of 13%, with 63% stabilizations [24]. In a retrospective analysis that we conducted in 17 patients with thyroid cancer of different histologies treated with sunitinib for advanced disease, we found an objective reduction of the size of metastases in 4 (33%) of the evaluable patient, and a median PFS of 13.3 months in the overall patient groups, 8.6 months being the PFS in those patients who had failed to one prior systemic treatment with sorafenib. The main grade 3-4 toxicities found in the study were asthenia (7.1%), thrombocytopenia (7.1%), and mucositis (5.9%) [34].

#### 2.4.5. Lenvatinib (E7080)

Lenvatinib (E7080) is a potent inhibitor in the nanomolar range of VEGFR-1 and -2 as well as PDGFR-*β* and the fibroblast growth factor receptor-1 (FGFR-1) (Table 1). In an international, open, phase II study which treated a total of 99 patients who had advanced thyroid cancer, lenvatinib showed an objective reduction in the size of metastases (according to RECIST criteria 1.1) in 29 (50%) of the 58 patients with histology of DTC. Also, 21 (36%) patients achieved disease stabilization, and the median PFS in all DTC patients was 12.6 months [25]. Among the 41 (70%) patients who had not received prior antiangiogenic therapy for advanced disease, the radiological objective response rate was 54% while, in the 17 patients who had previously received anti-VEGF, the observed partial response rate was 41%. However, dose reduction was required in 35% of patients, and 23% of them discontinued treatment due to toxicity. The most frequent grade 3 or 4 toxicities that led to dose reductions were hypertension (10%), proteinuria (10%), decreased weight (7%), diarrhea (10%), and fatigue (7%). Based on these promising data, we began a phase III registration study in which 360 patients with DTC refractory to radioactive iodine, which had been previously treated with other anti-VEGF agents, were randomized to receive lenvatinib or placebo. PFS was the primary aim of the study (NCT01321554).

#### 2.4.6. Motesanib

Motesanib (AMG706) is an oral inhibitor of multiple kinases, including VEGFR-1, -2, and -3 as well as the wild and mutant forms of the membrane receptor RET. In one of the initial phase I studies, focusing on recommended doses and major toxicities, motesanib was shown to achieve objective radiographic partial response in 3 of the 5 patients with thyroid cancer. This activity was not expected in these patients and led on to a prospective phase II study in 93 patients with DTC being carried out. One in every 3 patients remained on treatment for 48 weeks after commencement. The most common adverse events found at any grade were diarrhea (59%), hypertension (56%), fatigue (46%), and weight loss (40%). The radiological response rate assessed by independent reviewers was 14%, and up to 35% of patients benefited from a stabilization of their disease beyond 6 months. The median PFS was 9.3 months and, although the drug does not inhibit BRAF, the investigators observed that patients carrying a mutation in this pathway were less likely to progress if they received motesanib than patients without the mutation This finding suggested a greater involvement of angiogenesis in patients with the BRAF mutation [26].

#### 2.4.7. Pazopanib

Pazopanib shares the main targets with the other agents discussed above. However, it appears to have a marginal activity on *RET/PTC* and BRAF that is, its activity in these tumors is due mainly to its antiangiogenic effect rather than any intrinsic antitumor effect (Table 1). In a phase II study, pazopanib administered at a dose of 800 mg/day, induced a radiographic response rate of 49% in 37 patients with DTC who had disease progression over the previous 12 months. This response rate is among the highest achieved by any multitarget agent reported in this context. Up to 66% of patients who achieved a positive objective radiological response rate were able to maintain the response one year from the commencement of treatment. The median PFS in the total patient sample was 11.8 months. The most frequent toxicities found were fatigue (78%), skin rash (75%), diarrhea (73%), and nausea (73%). Contrary to that observed with axitinib, patients treated with pazopanib received the drug for a median of 12 months [27].

## 3. Medullary Thyroid Cancer (MTC)

### 3.1. Clinical Management of Patients with MTC

Currently, there were no officially approved therapy for the treatment of metastatic MTC until the recent approval of vandetanib by the American Food and Drugs Administration (FDA) and the European Medicine Agency (EMA). Following the failure of initial treatment by thyroidectomy and cervical lymphadenectomy, patients have received only symptomatic care measures. Therefore, this tumor has represented, and currently represents, a niche for the development of new active drugs, especially considering that between 35 and 50% of patients diagnosed with metastatic disease are likely to be diagnosed as having lympho-ganglionar metastases, and up to 15% of patients will develop distant metastases after diagnosis. Half of the patients diagnosed with MTC will ultimately present distant metastases during the clinical evolution of the disease.

MTC is considered a neuroendocrine tumor caused by the uncontrolled proliferation of para-follicular C cells producing calcitonin derived from the neural crest. It accounts for between 4 and 10% of thyroid cancers. In up to 30% of cases, the CMT is associated with an inherited syndrome and multiple endocrine neoplasia type 2 (MEN 2A or 2B) or with familial MTC (FMTC). Most hereditary MTC occur within MEN 2A that is often associated with pheochromocytoma or hyperparathyroidism [35].

Sporadic MTC is diagnosed in the 5th or 6th decade of life, while the hereditary versions of the disease can be diagnosed in 5-year-old children. Conventional therapy with chemotherapy has not proven to be particularly useful in patients with metastatic MTC. The most commonly used chemotherapy regimen has traditionally been that of doxorubicin monotherapy. This treatment achieves a clinical benefit, including the partial responses plus stable disease of 6 months, in about 21% of patients, with 79% of patients progressing within 5 months of treatment [27]. Regimens for neuroendocrine tumors have also been used for MTC, including a combination of streptozocin, dacarbazine, and 5-fluorouracil. Up to 30% of patients achieved objective response rates in small nonrandomized studies [36].

### 3.2. Molecular Biology of MTC (Figure 1)

Approximately 50% of patients with sporadic MTC have a somatic mutation at codon 918 of the *RET* gene. These mutations are considered “gain of function” type. The presence of this mutation is also associated with a high probability of lympho-ganglionary metastases, recurrent/persistent disease, and decreased survival. By contrast, germinal mutations at different loci of the *RET* gene are present in virtually all hereditary syndromes associated with MTC. There is a strong genotype-phenotype correlation between the location of the mutation in *RET* and the associated MEN2 type. This enables the patients to be classified into different risk groups according to the mutation they carry and as such, to be referred (or not) for prophylactic thyroidectomy [35, 37].

The abnormal activation of *RET* by any of the several mutations affecting the coding part of the gene can trigger activation of multiple intracellular signaling pathways such as RAS/RAF/MAPK, PI3K/Akt/mTOR, or Rac/JNK.

### 3.3. Multitarget Agents in Clinical Development of MTC (Table 3)

#### 3.3.1. Vandetanib

Vandetanib is the first drug approved in the US by the FDA and in Europe by the EMA for treatment of MTC in the last 30 years. The approval was as a consequence of the data reported in the ZETA study which was a phase III, double-blind trial with 2 : 1 randomization of patients to receive vandetanib at doses of 300 mg/day versus placebo. Between December 2006 and November 2007, a total of 331 patients with sporadic or inherited MTC were enrolled. The median PFS in the placebo arm was 19.3 months while the vandetanib treatment arm has not yet been concluded (HR: 0.46; 95% CI: 0.31–0.69; *P* < 0.0001) after 24 months of followup. The objective radiological response rate was also higher in the vandetanib treatment arm (45% versus 13%; *P* < 0.0001). Patients receiving vandetanib showed a statistically significant delay in time-to-pain worsening compared with patients receiving placebo (7.85 versus 3.25 months, HR: 0.61; 95% CI: 0.43–0.87; *P* = 0.006). Patients receiving this agent also showed significant decreases in serum concentrations of calcitonin and carcinoembryonic antigen (CEA). Adverse effects found were asthenia, weight loss, diarrhea, mucositis, hand-foot syndrome, and hypertension, most of them of grades 1 and 2 severity. Very rarely, some grade 3/4 events were observed. Interestingly, those patients harboring M918T mutation in *RET* had higher objective response rate (54.5%) than those with *RET *mutation unknown (34.1%) or negative (0%) [28].

#### 3.3.2. Cabozantinib (XL184)

Cabozantinib is an oral inhibitor that, in addition to acting against VEGFR-1 and -2, and c-KIT as are most of the drugs discussed above, is able to inhibit c-MET which, as also discussed above, is amplified in a large proportion of patients with DTC and MTC. It also acts against RET and against *RET/PTC* rearrangements (Table 1). In the extension phase of a phase I study containing 37 patients with MTC, we observed that 17 (49%) of 35 evaluable patients achieved an objective radiological response that, together with the 15 (41%) patients achieving stable disease over 6 months, totaled an overall clinical benefit of 90% of patients [29]. However, no significant correlation was observed between the presence of mutations in *RET* (in both sporadic and hereditary cases) and tumor response to cabozantinib. A phase III study comparing cabozantinib and placebo in patients with metastatic MTC is currently recruiting patients (NCT00215605).

#### 3.3.3. Sorafenib

The observed *in vitro* activity of sorafenib in MTC cell lines has stimulated investigators to design a phase II study in patients with hereditary (*n* = 5) and sporadic (*n* = 16) MTC. Analysis of the M918T mutation of the *RET* gene was performed in 12 of these patients, with 8 showing positive for the mutation. Preliminary results of the activity of sorafenib as a single agent in patients with MTC show that one (6%) patient achieved a partial positive response and 10 (62%) of 16 patients with sporadic MTC obtained a stabilization of their disease for >6 months. Serum levels of calcitonin and CEA decreased by over 62% and 44%, respectively [30].

#### 3.3.4. Motesanib

In a multicentered, international, open-label phase II trial, motesanib demonstrated that it was able to induce a clinical benefit in 50% of 91 patients with locally advanced or metastatic MTC. The PFS was 12 months. Most study patients (84%) corresponded to sporadic MTC in 72% of whom the *RET* mutation was present. There were no significant differences between the effectiveness of motesanib in patients in relation to the presence or absence of the *RET* mutation [31].

#### 3.3.5. Sunitinib

In the study by Carr et al. [23] that measured metabolic activity level in MTC patients, sunitinib showed a metabolic response (as measured by FDG uptake analyzed by PET) in the 3 of 8 patients in whom this measurement was performed. Subsequently, a phase II study specific for patients with MTC with disease progression over the previous 6 months was undertaken. Patients were treated with sunitinib at doses of 50 mg/day for 4 weeks followed by 2 weeks off drug. A total of 25 patients with MTC were recruited into the study. A partial response rate of 35% was achieved, with median response duration of 37 weeks; 57% of patients had stable disease as best response to treatment [32]. In patients with *RET* mutation and treated with sunitinib, the probability of remaining progression-free at one year was 88% (95% CI: 43%–98%).

## 4. Anaplastic Thyroid Cancer (ATC)

### 4.1. Clinical Management of Patients with ATC

ATC contributes less than 2% of all thyroid-derived tumors but is responsible for 14 to 39% of deaths from thyroid cancer. With a frequency 5-fold higher in men than in women, the median survival range depends on status between 3 and 9 months after diagnosis. Approximately 50% of patients diagnosed with ATC already have metastases at the time of diagnosis, and another 25% will eventually develop metastases. Treatment options are surgery, palliative radiotherapy, and chemotherapy. ATC tumors are considered chemo resistant. Doxorubicin in monotherapy achieves positive radiological response rates in <20% of patients, and this response is rarely protracted. Other platinum-based combinations such as taxanes, gemcitabine or vinorelbine can achieve up to 53% radiological response but does not have much impact on the overall survival of patients [38].

### 4.2. Molecular Biology of ATC (Figure 1)

Most frequent mutations observed in ATC affect* p53* gene in up to 70% of patients. Genomic aberrations of ATC also include RAS and *β*-catenin mutations in more than 50% of cases, and mutations in the oncogene *BRAF* have only been observed in around 20% of patients. Different genomic profiles between DTC or MTC and ATC probably are related with the higher aggressiveness of these tumors. Similarly, histological findings indicate high vascularity with overexpression of VEGF, the levels of which appear to correlate with stage and tumor size, lymph node, and distant metastases. Finally, EGFR is overexpressed in most of the patients with ATC. The implication of overexpression of EGFR on the clinical and biological behavior of these tumors is not known [38].

### 4.3. Multitarget Agents in Clinical Development in ATC

#### 4.3.1. Axitinib

In a phase II study conducted with single-agent axitinib in patients with thyroid cancer of all histology subtypes, there was an objective radiological response in approximately 50% patients with ATC who entered the study [22].

#### 4.3.2. Sorafenib

In the only study performed specifically in patients with ATC, 16 patients were treated with sorafenib alone. The rate of control of the disease was 40%, although time-to-progression barely reached 1.5 months. Of the 15 evaluable patients, 2 (13%) showed an objective radiological response that was maintained for a median of 5.3 months. The most frequently observed grade 3 and 4 adverse events were lymphopenia (31%), skin rash (12%), weight loss (12%), and chest pain (12%) [39].

#### 4.3.3. Combretastatin A4 Phosphate (CA4P)

CA4P is a vascular-disrupting agent, also called fosbretabulin tromethamine or combretastatin A4 phosphate. Unlike other antiangiogenesis agents that block tumor blood vessel formation, CA4P prevents blood flow in existing vessels. This drug has shown some activity as a single agent in patients with metastatic ATC who have not responded to at least one prior systemic therapy but in whom, on rare occasions, positive responses were observed and objective radiological PFS of 3 months was achieved. The final results in overall survival of the largest randomized trial conducted in patients with metastatic ATC were presented at American Society of Clinical Oncology (ASCO) annual meeting in 2011. A total of 80 of the planned 180 patients with ATC were recruited and randomized to receive carboplatin + paclitaxel with or without associated CA4P in a 2 : 1 randomization. The median overall survival was higher in the CA4P arm but did not reach statistical significance (5.2 versus 4.0 months; HR 0.65; 95% CI: 0.38–1.10); 27% of patients were alive at 1 year in the arm that received the experimental therapy compared to 9% in the standard treatment arm. The planned total patient sample could not be reached due to low recruitment [40].

## 5. Conclusion

Thyroid cancers are one of the greatest challenges for those treating advanced tumors. Thyroid cancer offers extensive opportunities to work in interdisciplinary teams that include not only oncologists and endocrinologists but also specialists in pathology, nuclear medicine, surgery, otolaryngology, radiology, clinical laboratory, and radiotherapy. Coordinated administration (whether concomitant or sequential) of the different therapeutic options for these patients make collaborative and efficient functioning of surgical and medical professionals more important than ever. The opportunity that presents itself is to apply the various treatments becoming available based on the genotypic characteristics of patients. Moreover, we have to consider the possibility of administering tyrosine kinase inhibitors for a long time with caution. We assume that these drugs are quite specific cytostatic and not cytotoxic agents, therefore, there is a need to give these agents until progression of the disease or toxicity. The concept of long-term treatment management of these new agents to patients in a childbearing age, with known cardiovascular toxicity, and perhaps many different side effects that could arise in the long-time followup, makes the monitoring of patients receiving inhibitors tyrosine kinase a must to be done very closely.

Maximizing clinical outcomes of tyrosine kinase therapy requires clear, effective communication, anticipation of side effects, and early intervention to avoid treatment delays and dose-limiting toxicities. There is a direct correlation between the plasmatic levels of the tyrosine kinase inhibitors with the activity of the drug, therefore, we would need to maintain as far as we can the highest dose of the targeted agent we consider is the most appropriate to our patients. In this sense, the experience in the management of tyrosine kinase inhibitors is crucial and probably the development of therapy management guidelines would be necessary in the next years.

To summarize, there is a need for a greater understanding of the molecular basis of the disease by the various health professionals charged with the care of these patient that could lead to a better optimization of treatment options in the advanced setting.

## Figures and Tables

**Figure 1:**
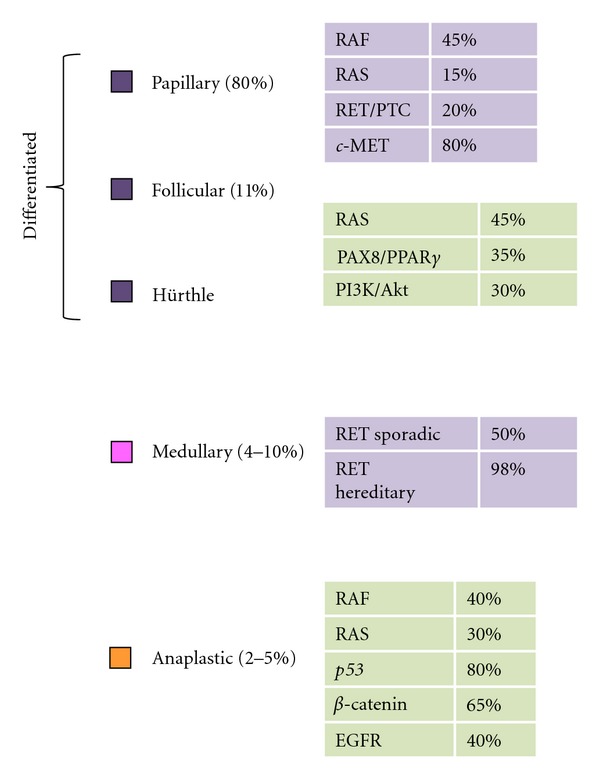
Molecular alterations present in different histological types of thyroid cancer.

**Table 1:** Inhibitory concentration 50 *(IC_50_)* of the major pharmaceutical compounds in clinical development for the treatment of thyroid cancer segregated with respect to the kinase activity required to inhibit different molecular targets (nmol/L).

Pharmaceutical compound	VEGFR1	VEGFR2	VEGFR3	RET	RET/PTC	PDGFR*β*	BRAF	KIT	Others (IC_50_)
Sorafenib	26	90	20	47	50	57	25	68	—
Motesanib	2	3	6	59	—	84	—	8	—
Axitinib	0.1	0.2	0.29	1.2	—	2	—	1.7	—
Sunitinib	10	10	10	100	224	39	—	1–10	—
Vandetanib	—	40	110	130	100	—	—	—	EGFR (500)
Pazopanib	10	30	47	—	—	84	—	74	—
Lenvatinib (E7080)	22	4	5.2	35	—	39	—	—	FGFR1 (46)
Cabozantinib (XL-184)	—	0.035	—	4	—	—	—	—	C-MET (1.8)

**Table 2:** Clinical data from studies with the main agents in clinical development in differentiated thyroid cancer.

Author (ref)	Pharmaceutical compound	*N*	Response rate (%)	Stabilizations (%)	Progression-free survival (months)
Gupta-Abramson et al. [16]	Sorafenib	30	23	53	20
Kloss et al. [17]	Sorafenib	41	15	56	15
Ahmed et al. [18]	Sorafenib	34	20 (DTC)	48	12
Hoftijzer et al. [19]	Sorafenib	31	24	34	14
Capdevila et al. [20]	Sorafenib	34 (16 DTC)	19 (DTC)	50 (DTC)	13.5
Leboulleux et al. [21]	Vandetanib	145	8.3 versus 5.5	48 versus 37	11 versus 5.8
Cohen et al. [22]	Axitinib	60	30	38	18
Carr et al. [23]	Sunitinib	33	13 (DTC)	68 (DTC)	12.8
Cohen et al. [24]	Sunitinib	31	13	63	Not reported
Sherman et al. [25]	Lenvatinib	56	47 (DTC)	36 (DTC)	Not reported
Sherman et al. [26]	Motesanib	93	24	67	10
Bible et al. [27]	Pazopanib	37	49	46	11.8

**Table 3:** Clinical data from studies with the main multi-target agents in clinical development for the treatment of medullary thyroid cancer.

Author (ref)	Pharmaceutical compound	*N*	Response rate (%)	Stabilizations (%)	Progression-free survival (months)
Wells Jr. et al. [28]	Vandetanib	231	45	—	Not achieved at 24 months. HR versus placebo = 0.46 *P* < 0.001
Kurzrock et al. [29]	Cabozantinib	37	49	41	Not reported
Lam et al. [30]	Sorafenib	16	6	62	17.9
Capdevila et al. [20]	Sorafenib	34 (15 MTC)	47	40	10.5
Schlumberger et al. [31]	Motesanib	91	2	48	11.2
Carr et al. [23]	Sunitinib	7	37.5	—	Not reported
De Souza et al. [32]	Sunitinib	25	35	57	Not reported
